# Culturally adaptive storytelling intervention versus didactic intervention to improve hypertension control in Vietnam- 12 month follow up results: A cluster randomized controlled feasibility trial

**DOI:** 10.1371/journal.pone.0209912

**Published:** 2018-12-31

**Authors:** Hoa L. Nguyen, Duc A. Ha, Robert J. Goldberg, Catarina I. Kiefe, Germán Chiriboga, Ha N. Ly, Cuong K. Nguyen, Ngoc T. Phan, Nguyen C. Vu, Quang P. Nguyen, Jeroan J. Allison

**Affiliations:** 1 Department of Non-Communicable Diseases, Institute of Population, Health and Development, Hanoi, Vietnam; 2 Department of Quantitative Sciences, Baylor Scott & White Health, Dallas, Texas, United States of America; 3 Cabinet Office, Ministry of Health, Hanoi, Vietnam; 4 Department of Quantitative Health Sciences, University of Massachusetts Medical School, Worcester, Massachusetts, United States of America; 5 Quantitative Biomedical Sciences Program, Dartmouth College, Hanover, New Hampshire, United States of America; TNO, NETHERLANDS

## Abstract

**Introduction:**

Vietnam is experiencing an epidemiologic transition with an increased prevalence of non-communicable diseases. The country needs novel, large-scale, and sustainable interventions to improve hypertension control. We report the 12 month follow-up results of a cluster randomized feasibility trial in Hung Yen province, Vietnam, which evaluated the feasibility and acceptability of two community-based interventions to improve hypertension control: a “storytelling” and a didactic intervention.

**Methods:**

The storytelling intervention included stories in the patients’ own words about coping with hypertension and didactic content about the importance of healthy lifestyle behaviors in controlling elevated blood pressure levels. The didactic intervention included only didactic content, which were general recommendations for managing several important risk factors for hypertension and other non-communicable diseases. The storytelling intervention was delivered by two DVDs three months apart; the didactic intervention included only one DVD. The trial was conducted in patients with poorly controlled hypertension from 4 communes (communities), which were equally randomized to the two interventions.

**Results:**

The mean age of the 160 patients was 66 years and 54% were men. Between baseline enrollment and the 12 month follow-up, mean systolic blood pressure declined by 10.8 mmHg (95% CI: 6.5–14.9) in the storytelling group and by 5.8 mmHg (95% CI: 1.6–10.0) in the didactic content group. The storytelling group also experienced more improvement in several health behaviors, including increased levels of physical activity and reduced consumption of salt and alcohol.

**Conclusions:**

We observed considerable long-term beneficial effects of both interventions, especially of our storytelling intervention, among patients with inadequately controlled hypertension. A large scale randomized trial should more systematically compare the short and long-term effectiveness of the two interventions in controlling hypertension.

**Trial registration:**

ClinicalTrials.gov: NCT02483780.

## Introduction

The morbidity and mortality from non-communicable diseases (NCDs) has been increasing in Vietnam over the last two decades and [1, 2] the changing profile of chronic disease in Vietnam parallels changes in the socio-demographic and lifestyle characteristics of the population and increases in life expectancy.[1–5] In Vietnam, NCDs account for an increasing proportion of all deaths, with cardiovascular disease (CVD) now being the leading cause of death, and hypertension is one of the major predisposing conditions leading to these elevated death rates.[2, 6]

Despite ongoing economic hardships in Vietnam, inexpensive health care, including generic medications to treat high blood pressure, are readily available, though we showed previously that only one-third of individuals diagnosed with hypertension in Thai Nguyen province were aware of their condition and, of those diagnosed with hypertension, only 43% were treated.[7]

We have developed two different interventions to promote engagement in the care of individuals diagnosed with hypertension in rural settings of Vietnam: use of a didactic content digital video disc (DVD) versus a culturally sensitive storytelling intervention.[8, 9] We have previously reported on the feasibility and acceptability of these two blood pressure lowering strategies over a 3-month follow-up in the context of a pilot randomized trial. We now provide data about our long-term, 12 month, follow -up results with regards to the potential effects of the two interventions on blood pressure control and on important risk factors for NCDs.

## Methods

The protocol for this trial and supporting CONSORT checklist are available as supporting information; see S1 Checklist and S1 Protocol.

Standard operation procedures were developed at the beginning of the study and health care workers and study personnel were carefully trained in specific aspects of the study protocol and in carrying out standardized measurement procedures, as described in detail previously. [8, 9]

### Trial setting

A detailed protocol of this feasibility trial has been published previously.[8, 9] In brief, this trial was conducted in Hung Yen province in northern Vietnam, which has a population of nearly 1.2 million people, organized into 10 districts and 161 communes. Four communes, namely Xuan Quan, Viet Hung, Tan Viet and Bach Sam located in four districts in this province were chosen for this trial based on their general representativeness to persons living in rural settings of northern Vietnam. Each of the selected communes had to have a community health center with a physician, were not previously or currently participating in other studies to improve the management of risk factors for CVD, and had a minimum geographic separation of 12 kilometers (7 miles) from other study communes to minimize possible contamination of the trial interventions.

### Storytelling intervention development

As described in detail previously, [8, 9] the development of the storytelling intervention was culturally adapted for patients with hypertension in Vietnam. This tailored intervention was built on successfully used protocols developed by our team in the United States for story elicitation, review, editing, and packaging;[10] however, all content for this intervention was based on discussions with patients residing in our participating study communes who spoke naturally about their hypertension and their personal and cultural beliefs.[8, 9] Patients were encouraged to comment on and share their experiences about family’s and neighbor’s support or challenges when they decided to modify their lifestyle practices to better manage their hypertension. In addition, study participants were encouraged to share their personal stories about whether they used traditional medicines or practiced mindfulness (e.g., meditation) to manage their elevated blood pressure, in addition to taking prescribed antihypertensive medications, since these practices are commonly used in Vietnam.

“We Talk about Our Hypertension”- the storytelling intervention, centered on patient stories about living with hypertension with patients speaking in their own words. [8, 9] A total of six Story Development Groups were carried out whose group discussions were intended to: (1) gather critical data to inform the intervention content; (2) identify patients who would serve as our storytelling “stars;” and (3) develop customized interview guides for the subsequent videotaping of each star.

This intervention approach focused on the more optimal management of high blood pressure through the use of, and increased adherence to, antihypertensive medications as well as personal lifestyle modifications in which patients told their own stories about how they were able to manage their hypertension effectively. [8, 9] Digital video sequences were rated by a team of several community members, health care workers, and study personnel who viewed and rated the DVDs using a simple questionnaire for strength of content and personal engagement. The most highly rated patient stories were subsequently integrated into two interactive DVDs; each DVD had five stories in Vietnamese and was approximately 50 minutes in length. [8, 9]

The DVD domains centered around stories about the health consequences of hypertension, overcoming barriers to hypertension management, and the importance of adherence to prescribed antihypertensive medication and lifestyle changes [8, 9] Patients’ perceptions about the benefits and use of traditional and Western medicine for controlling hypertension were also discussed.

### Didactic intervention development

The patient stories were supplemented by didactic “Learn More” content, which was coordinated with specific patient stories to fill in gaps not covered by the storytelling stars.[8, 9]

The DVD for the didactic intervention (comparison group) included general recommendations for managing several important risk factors for hypertension and other NCDs including the importance of having a healthy diet, quitting smoking tobacco, drinking less or no alcohol, participation in regular physical exercise, and having regular examination checkups. [8, 9]

### Trial inclusion criteria

As described in previous publications, [8, 9] consenting adults 50 years and older from our 4 study communes with poorly or inadequately controlled hypertension needed to fulfill several socio-demographic and clinical criteria to be enrolled in the trial.

### Participant recruitment and randomization

The recruitment of participating communes and study subjects has been described in detail previously. [8, 9] In brief, in this cluster randomized controlled trial, the four communes were randomly assigned to either intervention (two communes) or comparison status (two communes) as trial randomization was done at the commune and not individual patient level. At the time of screening, the blood pressures of adults 50 years and older with uncontrolled or poorly controlled hypertension from the 4 communes were measured and study staff explained the study protocol to possible participants and verbal consent was obtained. A second screening visit was planned two weeks later, at which time blood pressure was re-measured and written informed consent was obtained from all patients. Participant recruitment was completed in December 2015.

### Intervention delivery

As described previously, [8, 9] after obtaining informed consent, a trained community health worker introduced and explained the DVD to the patient and instructed them on how to use it at home. Initially, patients viewed the first DVD installment at their community health center and then engaged in a post-media interview and problem-solving session with a community health worker. After the first clinic viewing, the patient took the DVD home for further review.

At three months after trial enrollment, a second installment of the DVD was delivered for viewing at home by patients assigned to the storytelling intervention. After viewing the second DVD, a follow up visit was scheduled for a “post-media” interview and re-measurement of blood pressure by a trained community health worker. Patients assigned to the didactic intervention group only received a single DVD at the time of trial enrollment.

### Patient follow—Up

As described previously, [8, 9] one week prior to the scheduled follow-up visit, community health workers contacted patients by either phone or through a home visit to remind them about their subsequent follow up visits. For patients who missed the follow- up visit, community health workers came to their homes to measure their blood pressure and conducted an interview within two weeks of their scheduled follow-up visit. To enhance patient participation, we provided study patients with a DVD player at no cost at the beginning of the study and an Omron blood pressure monitor at the end of the study. Participant follow-up was completed in December 2016.

### Data collection activities

Data were collected at the time of baseline trial enrollment and subsequent scheduled follow-up visits at the patients’ local community health center. [8, 9] Patients’ blood pressure levels were measured by trained study staff at each study visit according to a standardized protocol.[11] Three measurements of blood pressure were separated by at least one minute, and values from the last two measurements were averaged and entered into the database. Height, weight, and waist and hip size were also measured according to a standardized protocol.[11]

Trained research nurses at each of the local community health centers collected data on patient’s socio-demographic factors and risk factors for CVD including self-reported tobacco use, alcohol consumption, salt intake, and a validated measure of physical activity.[13–17]

### Trial outcomes

Our principal trial outcomes have been described previously. [8, 9] Feasibility outcomes included participant recruitment and rates of retention during the course of follow-up. Data on acceptability and intervention engagement were collected from the “post media” interview. This included the amount of time spent viewing the DVDs, satisfaction with the viewing experience, and whether the DVDs were user friendly, informative, reasonable in length, and effectively encouraged patients to modify their current lifestyle practices.

Our exploratory outcomes included patient’s levels of systolic and diastolic blood pressure and the proportion of patients with controlled hypertension as defined in Joint National Committee JNC 8 (<150/90 mmHg for those ≥ 60 years old, and <140/90 mmHg for those < 60 years old or with diabetes or chronic kidney disease). [12] Risk factors for CVD included self-reported tobacco use, alcohol consumption, salt intake, and extent of moderate and vigorous physical activity patients regularly undertook. (13) Number of hours spent on a weekly basis in physical activity of moderate and vigorous intensity were weighted by their Metabolic Equivalent Take (MET) values provided in WHO guidelines (moderate activity is assigned a Met of 4 and vigorous activity is assigned a Met of 8), and were classified into 2 categories using standard cutoffs: low (<600 MET- minutes), moderate or high (≥ 600 MET- minutes).[11]

### Data analysis

Categorical data were presented as frequency distributions and continuous variables were presented as medians (Inter quartile range-IQR).

Distributions of systolic and diastolic blood pressure were examined using histograms and univariate statistics. There was no missing data except for two individuals who did not have data at 12-month follow up. Distributions of systolic and diastolic blood pressure were skewed to the right, as would be expected for individuals enrolling in a hypertension study. However, all recorded values of blood pressure were biologically plausible.

To preserve the power of randomization, our modeling strategy was designed to test two primary null hypotheses that were developed before data were collected, namely that the study groups (intervention versus comparison) would manifest no difference in over-time change (baseline to 12 months) for systolic blood pressure (H1) or diastolic blood pressure (H2). Therefore, we used generalized linear mixed regression models which included group assignment, time of assessment, and the interaction between study group and time to estimate changes over time in selected study outcomes, accounting for clustering of patients within commune and repeated within-person measurements by random effects. As such, the intervention effect was parameterized by the interaction coefficient, which represented different over-time change for the intervention versus comparison group. Each patient contributed data at two time points (baseline and 12 months after trial enrollment) for analyses presented in this manuscript.

Because of the differences in baseline blood pressure measurement noted between the two study groups, secondary analyses added baseline blood pressure as a model covariate.[13] However, the main results for the intervention effect were not substantively changed.

The study dataset had two levels of clustering that were addressed by our analytic approach. First repeated blood pressure measurements were taken within participants, and, second, participants were clustered within communes (clinical sites). Random-effects models allowed us to appropriately model this hierarchical nature of the data and to examine the proportion of variance attributable to each level of clustering.[14] Random-effects relaxed the assumption that covariates are measured without error and accounted for the correlation of observations resulting from clustering with in a dataset, thus allowing the model to correctly estimate standard errors.

Therefore, the original analysis included two levels of clustering to appropriately represent the hierarchical nature of the data. First, there were repeated blood pressure measurements made within participants over time, and, second, participants were clustered within communes (clinical sites). However, because the models for systolic and diastolic blood pressure showed a vanishingly small fraction of variation in the outcome attributable to clustering at the commune level (intraclass correlation < 0.0001), we only entered the individual participant as a random effect for the final model. We found no graphical or statistical evidence that the models were influenced by extreme values or that the random-effect assumption was inappropriate.

Based on this final model with coefficients for group, time, and group-time interaction and with one level of random effects to account for repeated measurements within participants, we estimated post-regression marginal effects for change in blood pressure over-time. Standard errors for the marginal estimations were based on a second-order Taylor expansion series.[15]. All analyses were performed using STATA 15.1 (StataCorp. TX).

### Ethical considerations

As described previously, [8, 9], this randomized trial was approved by the Institutional Review Board at the University of Massachusetts Medical School (H00005592) and the Institute of Population, Health and Development in Hanoi, Vietnam (2014/PHAD/UMMS 01–01).[8, 9] Written informed consent was obtained from all patients. This trial was registered at ClinicalTrials.gov (Registration number: NCT02483780; registration date June 22, 2015).

## Results

### Study population characteristics

**Fig 1:**
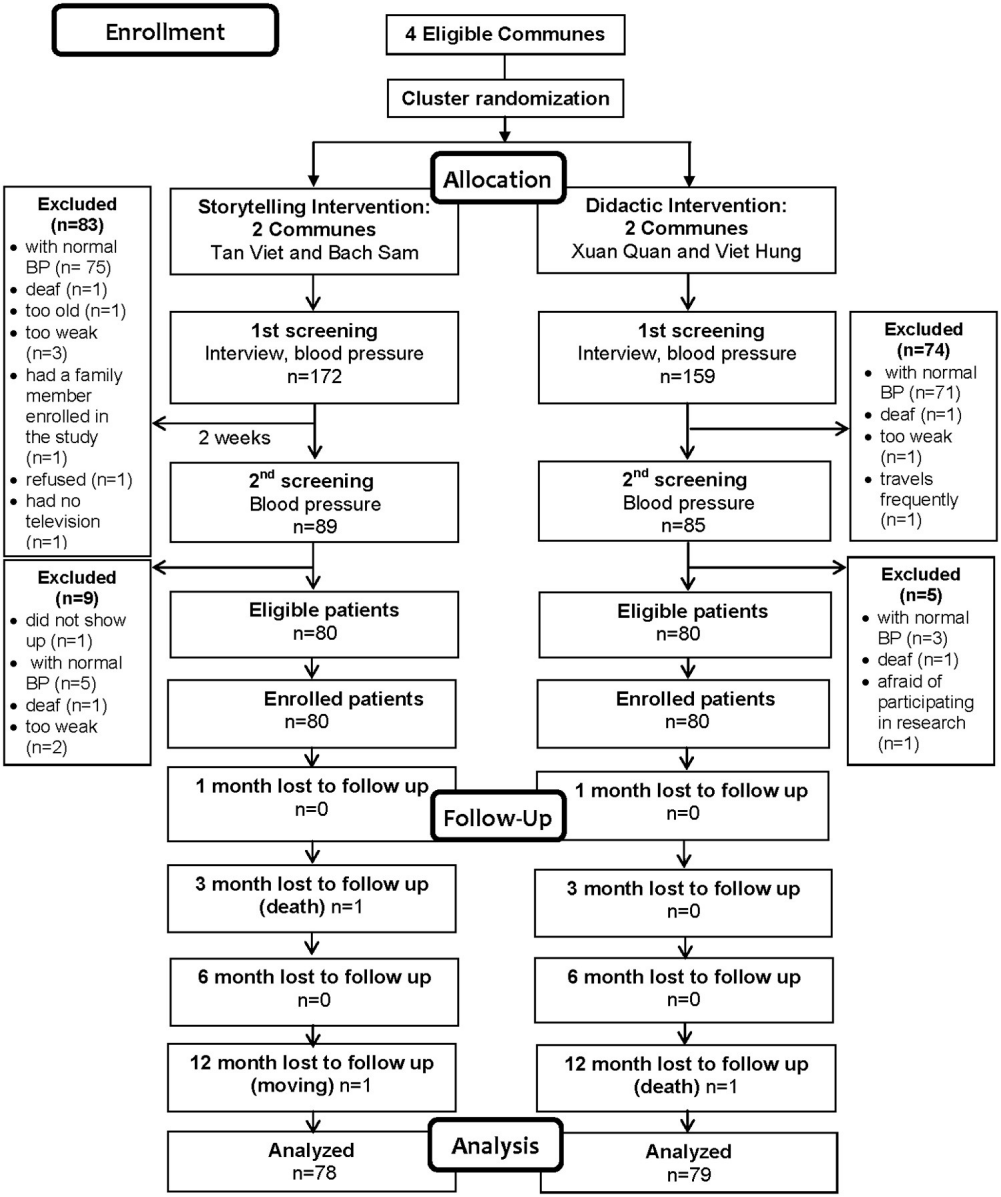
CONSORT flow diagram for the feasibility cluster randomized controlled trial.

Full details about the screening and follow-up process are shown in Fig 1. In brief, a total of 160 eligible patients (80 patients in each of the intervention and comparison groups) were identified and invited to participate in the trial. All eligible patients agreed to study participation and provided written informed consent. At 12 months after enrollment, 98% (157 out of 160) of study patients were successfully followed up.

The mean age of the study population was 66 years and 54% were men (Table 1). The blood pressure levels were significantly higher in the storytelling than in the didactic intervention group at the time of baseline trial enrollment. There were no important differences in other trial baseline characteristics for those randomized to either the storytelling or didactic intervention groups (Table 1).

**Table 1:** Patient characteristics at the time of trial enrollment according to intervention condition.

Patient characteristics	Storytelling(n = 80)	Didactic(n = 80)	P-value
Age (mean, SD), years	64.6(9.9)	66.9(8.9)	0.13
Body mass index (median-IQR), kg/m^2^	23.1(21.9–24.8)	24.0(21.7–25.5)	0.48
Number of adults residing in the household (median-IQR)	3(2–4)	3(2–4)	0.90
Systolic BP (SD), mean, mmHg	150.4 (17.5)	144.3 (8.9)	0.007
Diastolic BP(SD), mean, mmHg	91.3 (9.3)	87.8 (8.1)	0.011
	**N(%)**	**N(%)**	
Male	39(48.8)	47(58.8)	0.21
Ethnicity			
Kinh	80(100)	80(100)	NA
Marital status			
Never married	2(2.5)	0(0)	0.12
Currently married	64(80.0)	73(91.2)	
Widowed	14(17.5)	7(8.8)	
Level of Education			
Primary school	21(26.3)	24(30.0)	0.21
Secondary school	36(45.0)	38(47.4)	
High school	17(21.3)	7(8.8)	
College/University	6(7.4)	11(13.8)	
Working status			
Employee	3(3.8)	4(5)	0.56
Self-employed	12(15)	10(12.5)	
Homemaker	11(13.8)	8(10)	
Retired	16(20)	26(32.5)	
Other	38(47.4)	32(40)	
Current smoker	13(16.3)	12(15)	0.83
Self-reported CVD comorbidity			
Elevated blood glucose/diabetes	11(18.6)	12(21.8)	0.72
Elevated blood cholesterol	34(61.8)	27(52.9)	0.52
AMI/ angina/stroke	27(33.8)	30(37.5)	0.55
Controlled blood pressure*	13(16.3)	20(25.0)	0.17

SD: Standard Deviation, IQR: Interquartile range, BP: Blood pressure, CVD: Cardiovascular disease.

*Based on guidelines from the Joint National Commission for the Management of High Blood Pressure in Adults.

### Intervention acceptability and engagement

Intervention acceptability and engagement, and results from the survey about viewing the first DVD, have been published previously.[8] At the time of the 6-month in-person follow up visit, a survey about the viewing of the second DVD was administered to both study groups. In the storytelling intervention group, the median frequency of viewing the DVD on a weekly basis was 2 times (IQR: 1–4), and the median duration of time spent watching the DVD each time it was reviewed was 60 minutes (IQR: 25–60 minutes). More than one half of the patients in this group reported that they had watched all 5 “star” stories on the first DVD (Table 2). The majority of patients stated that they understood the words used in the storytelling DVD (96%) and that the stars’ stories were culturally acceptable (92%). The majority of patients also reported that the content of the DVD’s was image attractive (80%), informative (97%), reasonable in length (94%), and effectively encouraged them to change their lifestyle practices (76%).

**Table 2:** Post second DVD viewing survey according to intervention condition.

Responses	Storytelling	Didactic
Median (IQR) frequency of viewing DVD per week	2(1–4)	1(1.0–2.0)
Median (IQR) duration of viewing DVD each time (minutes)	60(30–60)	12(12–12)
Viewed all 5 stories (n,%)	46(58)	NA
Understandable words (n,%)	75(96.2)	76(95.0)
Culturally acceptable (n,%)	72(92.3)	79(98.8)
Attractive images (n,%)	60(76.9)	60(75.0)
Telling story/health topic attractively (n,%)	62(79.5)	63(78.8)
Encouraged to change habits (n,%)	59(75.6)	69(86.3)
Length (n,%)		
Reasonable	73(93.6)	27(33.8)
Too long	5(6.4)	2(2.5)
Too short	0(0)	51(63.7)
Information (n,%)		
Enough	76(97.4)	51(63.7)
Too much	2(2.6)	0(0)
Not enough	0(0)	29(36.3)

DVD: Digital video disc; IQR: Interquartile range; NA: Not applicable

For the didactic intervention group, the median frequency of viewing the DVD on a weekly basis was 1 time (IQR: 1.0–2.0), and the median duration of viewing the DVD was 12 minutes (IQR: 12–12 minutes). Most of the patients in this group stated that the didactic instruction DVD was image attractive and effectively encouraged them to change their heart healthy lifestyle practices (Table 2). More than one half of the subjects in this group reported that the DVD was too short, and more than one-third stated that that were was inadequate information presented about how they might better control their blood pressure.

### Intervention effects on blood pressure levels

The average systolic and diastolic blood pressure readings improved over time for both study groups (Table 3). However, greater reductions in average blood pressure levels were consistently noted for persons assigned to the storytelling versus the didactic intervention.

**Table 3:** Improvement Over-time (95% CI) in Blood Pressure Findings after Exposure to Storytelling and Didactic Interventions^§^.

Study Outcomes	Storytelling	Didactic
Systolic Blood Pressure (mean, SD), mmHg		
Baseline	150.4 (17.5)	144.3 (8.9)
Month 12	139.7(19.4)	138.5(16.7)
Δ Baseline—Month 12	10.7(6.5–14.9)	5.8(1.6–10.0)
Diastolic Blood Pressure(mean, SD), mmHg		
Baseline	91.3 (9.3)	87.8 (8.1)
Month 12	84.7(9.8)	84.1(12.5)
Δ Baseline—Month 12	6.6(3.9–9.3)	3.8(1.1–6.4)
Controlled Blood Pressure (%)*		
Baseline	16.3	25.0
Month 12	47.4	45.6
Δ Month 12- Baseline	31.4(18.4–44.3)	20.5(7.2–33.7)

SD: Standard deviation; CI: Confidence interval.

^§^Based on generalized linear models that account for clustering of patients within commune and repeated measurement nested within patients. Small discrepancies from obtaining difference directly are attributable to missing data and numerical estimation algorithms.

*Based on guidelines from the Joint National Commission for the Management of High Blood Pressure in Adults.

At 12 months after trial randomization, patient’s mean systolic blood pressure had declined by 10.7 mmHg (95% CI: 6.5–14.9 mmHg) in the storytelling intervention group and by 5.8 mmHg (95% CI: 1.6–10.0 mmHg) in the didactic intervention group. Similarly, patient’s mean diastolic blood pressure had declined by 6.6 mmHg (95% CI: 3.9–9.3 mmHg) in the storytelling intervention group and by 3.8 mmHg (95% CI: 1.1–6.4 mmHg) in the didactic intervention group. Rates of blood pressure control improved over time for both groups after exposure to the trial interventions (Table 3).

### Intervention effects on important risk factors for CVD

At 12 months after trial randomization, the rates of self-reported abstinence from smoking, drinking alcohol less often, and participation in moderate and vigorous physical activity improved for both groups over time, with greater improvements noted in the storytelling group (Table 4).

**Table 4:** Improvement Over-time (95% CI) in Risk Factors for Cardiovascular Disease (CVD) after Exposure to Storytelling and Didactic Interventions^§^.

CVD Risk Factors	Storytelling (n = 80)	Didactic (n = 80)
BMI, kg/m2 (mean, SD)		
Baseline	23.5(2.8)	23.6(2.7)
Month 12	23.6(2.9)	23.6(3.0)
Δ Baseline—Month 12	0.05(-0.19–0.30)	-0.08(-0.32–0.16)
Current smoking (%)		
Baseline	16.3	15.0
Month 12	9.0	11.4
Δ Baseline—Month 12	9.7(-1.6–20.9)	6.8(0.1–13.4)
Drink alcohol ≥ 5 days last week (%)		
Baseline	13.8	12.5
Month 12	7.7	10.1
Δ Baseline—Month 12	6.5(1.0–12.1)	2.7(-4.8–10.1)
Always/often add salt or salty sources to food (%)		
Baseline	66.7	70.9
Month 12	63.9	70.6
Δ Baseline—Month 12	2.8(-0.12–0.18)	0.3(-14.5–15.1)
Low physical activity (<600 MET-mins)* (%)		
Baseline	27.5	21.3
Month 12	10.3	11.4
Δ Baseline—Month 12	17.2(7.0–27.3)	9.8(0–19.5)

CVD: Cardiovascular disease; CI: Confidence interval; BMI: Body mass index; SD: Standard deviation.

^§^Based on generalized linear models that account for clustering of patients within commune and repeated measurement nested within patients. Small discrepancies from obtaining difference directly are attributable to missing data and numerical estimation algorithms.

*Based on moderate and/or vigorous activities in a typical week.

## Discussion

Middle-aged and older adults with elevated blood pressure who reside in rural communities in Vietnam face unique challenges in managing their hypertension. In response to these challenges, and to be socio-culturally sensitive, we developed two literacy sensitive interventions with one intervention incorporating stories told in the patients’ own words and the other intervention relying totally on didactic content contained on a DVD. The results of our 12 month follow-up show considerable long-term beneficial effects of both interventions, especially of our story telling intervention, in lowering blood pressure and improving important lifestyle risk factors for hypertension and CVD in middle-aged and older Vietnamese men and women with hypertension.

The present results suggest that both the storytelling and didactic interventions were well received among residents of several rural communities in northern Vietnam, although patient ratings were consistently higher for the storytelling intervention. Most of the patients stated that the storytelling DVDs were understandable, culturally acceptable, informative, reasonable in length, and effectively encouraged them to modify any of their adverse lifestyle practices. Study protocols for randomization and data collection worked well, and were well accepted by patients and health care workers at local community health centers.

It is likely that the positive changes that we observed on patient’s heart healthy lifestyle behaviors may have had a favorable effect on lowering the blood pressure levels of this population. Indeed, we observed positive changes in current smoking, alcohol, and salt consumption, and in obtaining regular physical exercise in both intervention groups, with greater improvements in each of these risk factors observed in the storytelling group.

The present findings are consistent with a previous study carried out by our group among African Americans living in Alabama with hypertension which showed that exposure to a culturally sensitive storytelling intervention produced a significant lowering in blood pressure and control of their hypertension for patients treated at an urban, safety-net setting.[10, 12, 16]

To develop the Vietnam storytelling intervention that we used in this feasibility and acceptability study, we drew upon the conceptual underpinnings of our previous work in African Americans living in Alabama [12, 16] and adapted the specific intervention to the new cultural setting of rural Vietnam. Our conceptual model of storytelling holds that powerful stories told in the patients’ own words create emotional resonance as the viewer becomes open to change to various messages, including altering one’s negative lifestyle practices and increasing their adherence to prescribed medications.[17, 18] We believe that the present results support the notion that storytelling is a universally shared and valued human activity that offers numerous opportunities for effective health promotion, including favorable effects on hypertension control and other important risk factors for NCDs.

Despite the pilot nature of this feasibility trial, and the beneficial effects we observed of both trial interventions on patient’s blood pressure levels, control of hypertension, and modification of important lifestyle risk factors for CVD, there are several potential limitations that must be kept in mind in interpreting our study results. First, this pilot trial was intentionally not powered to compare changes over time in the levels of blood pressure between the two study groups because of the nature of its design. Second, there was the potential for contamination of the trial interventions despite the distance between adjacent communes being at least 12 kilometers (7 miles) apart. During the conduct of the study, it was possible that patients in the didactic intervention group also received information on CVD risk prevention at local community health centers and from the mass media, which may have contributed to their favorable outcomes. Furthermore, we were not able to rule out the fact that some of the improvements in blood pressure that we observed resulted from being enrolled and followed up in a clinical trial as opposed to representing a specific beneficial effect of the delivered intervention.

## Conclusions

The results of our one-year patient follow-up show considerable long-term beneficial effects of both interventions, especially of our storytelling intervention, in lowering blood pressure and improving the profile of important risk factors for NCDs among middle-aged and older adults living in rural Vietnam with hypertension. Moreover, each of these interventions was deemed to be highly acceptable and viewed favorably by our study population. The present trial findings suggest that the implementation of a subsequent large scale, fully powered, cluster-randomized controlled trial comparing the short and long-term efficacy of both interventions is an appropriate next step. The results of such an intervention trial would hopefully provide clinicians, researchers, and health policy makers with practical evidence on how to combat a major predisposing factor for CVD using a feasible, sustainable, and highly cost-effective intervention. The present work provides insights into how this approach can be incorporated into various health care and home settings and used to modify important risk factors for chronic diseases of major public health importance.

## Supporting information

S1 Checklist (DOCX)

S1 Protocol (PDF)
