# Supplementary material for: Culturally adaptive storytelling intervention versus didactic intervention to improve hypertension control in Vietnam- 12 month follow up results: A cluster randomized controlled feasibility trial
Source: PLoS One. 2018 Dec 31;13(12):e0209912. doi: 10.1371/journal.pone.0209912 (PMC6312314; doi:10.1371/journal.pone.0209912)
Supplement: S1 Protocol — (PDF) [file pone.0209912.s002.pdf]

S1 Protocol

Project Title

Chúng ta nói về bệnh Tăng huyết áp

We Talk About Our Hypertension

Institute of Population Health and Development

2014-2016

Supported by:

National Institutes of Health, Fogarty International Program

Award number: 1 R21 TW009740-01

Table of Contents

Summary 2
Specific Aims 3
Background and Rationale 4
Epidemiologic Transition and Cardiovascular Disease (CVD) 4
Hypertension and Its Impact 4
Conceptual Models 5
Trial design 7
Study Setting 7
Phase 1. Intervention Development 8
Phase 2. Randomized Trial Evaluating the Feasibility and Acceptability 9
Data Analysis Plan 15
Timeline 15
References 17

Summary

Study Objectives

We propose to develop a novel community-based intervention, using the “storytelling” method, to enhance the control of elevated blood pressure in adults residing in rural communities in the Red River Delta region of Vietnam and to evaluate the feasibility and acceptability of the intervention through the conduct of a cluster feasibility randomized trial.

Study Design and Trial Outcomes

A cluster feasibility randomized trial will be conducted in Hung Yen province, Vietnam to evaluate the feasibility and acceptability of a novel community-based intervention using the “storytelling” method to enhance the control of HTN in adults residing in 4 rural communities.

Feasibility outcomes include recruitment, retention, engagement, and treatment fidelity. Exploratory outcomes will include levels of systolic and diastolic BP and the proportion of participants with controlled BP defined as in JNC-8 at three months after randomization.

Trial Intervention

The intervention will center on stories about living with HTN, with patients speaking in their own words. The stories will be obtained from particularly eloquent patients, or “video stars” identified during Story Development Groups.

Study Population

Four eligible communes in 4 districts in Hung Yen province will be randomly assigned to the intervention group (n=2) or comparison group (n=2). Within each selected commune, 25 individuals 50 years or older with HTN will be enrolled in the trial resulting in a total sample size of 100 patients. Each of the selected communes satisfy the following criteria: (1) have a community health center with a medical doctor; (2) are not currently participating in other studies for hypertension control; and (3) have a minimum geographic separation of 12 kilometers (7 miles) from all other study communes to minimize possible contamination.

Patient Population

Individuals 50 years or older with uncontrolled HTN (n = 100) will be assigned to intervention versus comparison status based on the commune in which they reside.

Specific Aims

The main goal of the "Storytelling" intervention will be to promote engagement in preventive health care and lifestyle behaviors and improve the expected low rates of hypertension (HTN) control among adult residents of a rural province in Vietnam. The specific aims of our work are to develop and evaluate the feasibility and acceptability of a novel community-based intervention using the "storytelling" method to enhance the control of HTN in middle-aged and older adults residing in a rural province of Vietnam which will be accomplished in several overlapping phases.

Phase 1: Developmental Phase: Intervention development

Specific Aim 1: Engage community members from Hung Yen province, a rural province of Vietnam, to produce an interactive, multi-media intervention based on patients' stories in culturally and literacy appropriate ways.

Specific Aim 2**:** Produce a series of interactive DVDs that encompass health message domains which are consistent with the Health Behavior Model, the Adapted Slater Model of Narrative Communication, the cultural background of participants, and the desired behavior change.

Specific Aim 3: Use state-of-the art approaches to culturally adapt and translate intervention and assessment methods that have been successfully used in other settings and for other populations.

Phase 2: Implementation Phase: Feasibility trial

Specific Aim 4: Conduct a feasibility cluster-randomized controlled trial (RCT) with 4 communes randomly assigned to either an intervention or comparison condition. Feasibility

and acceptability outcomes will include participant engagement, recruitment, retention, intervention fidelity, feasibility of assessment procedures, and participant satisfaction.

Background and Rationale

Epidemiologic Transition and Cardiovascular Disease (CVD)

Vietnam is in an epidemiological transition. The overall morbidity and mortality from non-communicable diseases (NCDs) has been rising rapidly over the last two decades and is a major societal problem.[1] The changing profile of chronic disease in Vietnam parallels changes in the socio-demographic characteristics of the population and increases in life expectancy.[1-4] Increased life expectancy prolongs the life-course exposure to risk factors for cardiovascular disease (CVD), rendering the population more susceptible to diseases of the heart and circulation; CVD is now the leading cause of death in Vietnam, accounting for 25% of all deaths.[5] Concomitant with these trends, the major risk factors for CVD are either on the rise or at alarming levels in the general population. A national survey in eight Vietnamese provinces and cities found that the prevalence of hypertension (HTN) was 25% in persons 25 years and older, and it increased with advancing age (prevalence of HTN in persons 45-54 years and 55-64 years were 42% and 58%, respectively).[6] The Vietnam National Health Survey in 2002 estimated that, by 65 years of age, nearly one half of all Vietnamese men and women will have HTN.[7]

Hypertension and Its Impact

The World Health Organization (WHO) considers HTN to be one of the most important causes of premature mortality worldwide.[8] It is also one of the most preventable CVD risk factors; it can be easily detected and effectively treated according to evidence-based guidelines with low-cost drugs.[9] [10] In spite of the economic hardships that exist in Vietnam, inexpensive health care including generic medications to treat HTN are readily available. Our research team has, however, reported disconcerting results from a population-based survey of residents of Thai Nguyen province in 2011 in which only one

third of persons diagnosed with HTN were aware of their condition. Furthermore, of those diagnosed with HTN, only 43% were on treatment, and of those being treated for HTN, only 39% had achieved appropriate control.[11] Furthermore, in a rural district in Vietnam, 83% of participants who had high blood pressure (BP) were not aware of their HTN, and only 6% of those with HTN were being treated.[10] These findings flow not only from inadequate training of community health care workers in effective health communication and lack of necessary skills to communicate the diagnosis of HTN, and the importance of control measures, to the patient, but also from poor knowledge of the importance and health impact of HTN in the general population. Indeed, a recent survey in Vietnam showed that 70% of community health workers were unable to identify essential questions to be asked of a patient with HTN.[12] Likewise, a nationwide survey in 2007 found that only 23% of participants understood what were the major risk factors for CVD with men and women from rural areas having particularly poor knowledge about these risk factors.[7] These findings suggest a clear need for educating the Vietnamese population, especially middle-aged and older adults residing in rural areas, about CVD risk factors, the adverse health effects of HTN, and the development of acceptable and sustainable low-cost interventions to effectively prevent and treat elevated BP.

Conceptual Models

We urgently need novel, large-scale, and sustainable public-health interventions for detecting, treating, and controlling HTN in Vietnam. Narrative intervention, or “storytelling,” is a promising approach for engaging low-literacy populations in the treatment of their HTN. A previous randomized controlled trial of an interactive, multi-media storytelling intervention by our team documented a substantial benefit for BP control.[13] However, this work was carried out among inner-city African Americans in Birmingham, Alabama and some of the protocols used for this intervention may not directly apply to different settings. However, as storytelling is a central part of what makes us human, narrative interventions have the flexibility to undergo adaptation to achieve the best results in new cultural settings.

With this flexibility of design and approach, we are adapting our previous storytelling work for a rural Vietnam population. More specifically, we are seeking to develop a novel community-based intervention using the "storytelling" method to promote the control of HTN among middle-aged and older adults residing in rural communities in the Red River Delta region of Vietnam and to evaluate the feasibility and acceptability of the intervention with a feasibility cluster-randomized trial.

Described briefly, the intervention development process begins with Story Development Groups, consisting of small groups of patients who gather for a guided discussion. From these groups, particularly eloquent "video stars" are selected to tell their story, which may focus on the health consequences of uncontrolled HTN, overcoming barriers to HTN control, adherence to prescribed medication, and importance of dietary and lifestyle changes among other topics that connect the narrative directly with cultural practices that can have an effect in the control of HTN. The intervention team then develops customized interview guides, captures video footage, and packages the stories in an appropriate context along with supporting didactic material.

Storytelling is inherently culturally appropriate and well suited for populations across the literacy spectrum; it is especially well suited for populations with low health literacy.[14] Conceptually, our intervention rests on two models to empower hypertensive patients with the support of community health workers: the Health Belief Model[15] and the adapted Slater Model of Narrative Communication Theory (Figure 1).[16]

Figure 1. Adapted Slater Model of Narrative Communication

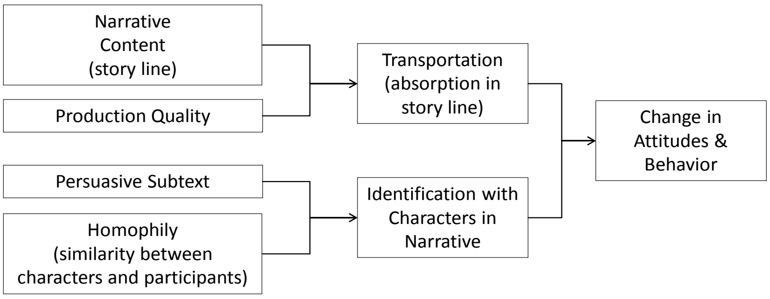

The Health Belief Model includes four constructs representing the perceived threat and net benefits of treatment including perceived susceptibility, disease severity, benefits of treatment, and barriers. The model posits that a person is more likely to take action or change their behavior when the perceived severity and susceptibility of the disease are high

and the balance between perceived barriers to changing behavior or treatment and benefits is favorable. Because HTN is primarily an asymptomatic, chronic condition that leads to serious morbidity and mortality if left untreated over several years, strategies for improving the rates of HTN control in the general population must address falsely held patient beliefs.[17-19] Our pilot data from Vietnam demonstrates a compelling lack of such awareness, and our previous work with storytelling suggests that our storytelling approach is an effective strategy for overcoming these barriers in a low-literacy population.

Narrative communication appeals to the human affinity for “storytelling.” Its effectiveness in changing attitudes and behavior follows from the ability to break down cognitive resistance through transportation (absorption in the story line) and identification with characters in the narrative. Thus, the participant is transported into the world of the characters, in this case hypertensive individuals, and becomes absorbed in the narrative content, rather than focusing on the embedded subtext of behavior change.[20, 21] The conceptual basis for the influence of stories on behavior derives from personal relevance, increased risk perception, increased self-efficacy, and transportation into the narrative (Figure 1).

Trial design

Study Setting

“We Talk About Our Hypertension” will be conducted in the Red River Delta Region which is an agriculturally rich and densely populated area in northern Vietnam. In this region, communes in Hung Yen province were selected based on their general representativeness. Hung Yen province has a population of approximately 1.2 million, organized into 10 districts and 161 communes. In Vietnam, the health system is organized into four levels, namely central, provincial, district hospital, and the lowest level, which includes the community health centers (CHCs) that are responsible for providing primary health care and outpatient services. Patients with HTN are typically treated and managed at the CHCs unless they need to be referred for a higher level of care.

All communes in Hung Yen have adequate electricity. Data from a national survey in 2010 showed that 94% of households in Hung Yen province have a television, 43% of households have landline phones, and 37% of the population uses mobile phones.[22] Each participant will receive a DVD player, which will be used to view DVDs, at no cost and will be given assistance in setting it up and also provided with ongoing troubleshooting.

Phase 1. Intervention Development

Intervention development will be based on successfully used protocols for story elicitation, review, editing, and packaging and tested in prior projects such as TRUST and Culturally Sensitive Intervention (CSI): Birmingham.[13] The intervention will center on stories about living with HTN, with patients speaking in their own words. The stories will be obtained from particularly eloquent patients, or “video stars,” identified during Story Development Groups. The power of this approach hinges on capturing authentic stories from real patients. In brief, the approach includes: (1) selection of “star” storytellers through Story Development Groups; (2) creation of a customized interview guide designed to elicit the most powerful stories for each star; (3) videotaping each star telling his or her story; (4) decomposition of videotaped interviews into “story units;” (5) rating of message strength for the story units by independent reviewers fluent in Vietnamese; (6) weaving the most highly rated video segments into a cohesive and authentic video production; and (7) piloting and “usability testing”[23] with multiple iterations and a “Thinking Aloud” Protocol.[24, 25]

The process will begin with six Story Development Groups to: (1) gather critical data to inform intervention content; (2) identify patients who will serve as our storytelling “stars”; and (3) develop customized interview guides for the subsequent videotaping of each star. Each Story Development Group will have between 6 and 8 participants with newly diagnosed or longstanding HTN. These HTN “stars” will have had positive experiences in controlling their HTN and be particularly eloquent and persuasive advocates.

“We Talk About Our Hypertension” will focus on how the stars in the videos manage their HTN using positive behaviors. Digital video sequences will be rated by

strength of content and emotional engagement according to our conceptual models based on adaptations of previously developed protocols from CSI: Birmingham.[13] The edited stories will be integrated into two interactive DVDs. Each DVD will have five stories on it and be about 30 minutes in length in total.

The content of the DVDs will focus on a series of health message domains which are consistent with the HBM, narrative communication, the cultural background of participants, and the desired behavior change. The domains are as follows: stories about the health consequences of HTN as a silent killer, overcoming barriers to HTN control, and importance of adherence to prescribed medication, quitting smoking, dietary changes, weight loss, reducing sodium/salt intake, increasing levels of physical activity, and moderate use of alcohol, patients' perceptions about the benefits of traditional medicine and Western medicine, and the use of traditional medicine in addition to Western medicine for managing HTN. Storytellers will discuss culturally and literacy specific aspects of patients' perceptions of HTN and HTN treatment and deal with the daily challenges presented by achieving HTN treatment goals within the broader context of the patient's life. In addition, the stories will be supplemented by more didactic "Learn More" content. The Learn More section will be coordinated with specific patient stories and will fill in gaps not covered by the storytellers. The Learn More section will include in lay language such topics as: What is HTN? What are the consequences of untreated HTN? How may HTN be treated without medications? What are some common medications used to treat HTN? How do these medications work and what are their side effects? Why is it important to take your medication even when you are feeling well? How should I speak to my doctor about high BP?

Phase 2. Randomized Trial Evaluating the Feasibility and Acceptability

We will conduct a feasibility cluster RCT with 100 adult hypertensive participants from 4 communes (25 participants from each commune) in Hung Yen province to assess the feasibility and acceptability of both the intervention process and the process of randomization and evaluation.

Study Sites

In consultation with the Chief Cabinet Officer of Hung Yen province, four communes in four separate districts with a total population of approximately 12,000 men and women have been selected for this feasibility study. With a national HTN prevalence rate among adults of about 25%, and over half of the total population being 25 years and older, the selected sites will provide enough persons with HTN for the proposed feasibility trial. Each of the selected communes have satisfied the following criteria: (1) having a CHC with a medical doctor; (2) not currently participating in other studies to improve CVD risk management; and (3) the distance between any 2 selected communes is more than 12 kilometers (7 miles) to minimize possible contamination.

Participant Eligibility

To be enrolled in this feasibility study, consenting adult men and women must fulfill each of the following criteria: (1) be a resident of the selected commune; (2) be aged 50 years or older; (3) have a diagnosis of HTN according to the 7th Joint National Commission of High Blood Pressure (JNC 7)[9]; (4) have BP measures consistently elevated at 2 time points (screening and 2 week after screening); (5) not be cognitively impaired (as assessed by study physicians); (6) not be a “story teller” who was used to develop the intervention; and (7) not be a family member of another participant in the study. We will restrict our patient population to patients aged 50 years or older since their prevalence of HTN is greatest, they are interested in storytelling, and they face unique challenges to managing their HTN.

Trained study nurses at study sites will explain to eligible persons about the study’ procedures and will obtain informed consent. Patients will be excluded if they are pregnant or unable or unwilling to provide informed consent.

Study Recruitment and Randomization

Four eligible communes will be randomly assigned to either the intervention or comparison condition. Participant assignment to respective study arms will be based on randomization status of their communes. This allocation program mirrors our plans for the

future, large-scale RCT. The population at risk will be screened for HTN because available health information systems do not allow us to obtain a sampling frame at the community level. To this end, sampling frames that comprise all adult community members will be obtained under the support of community collaborators. Based on the sampling frame, commune residents 50 years and older will be randomly selected for HTN screening. To minimize selection bias, enrollment of patients will proceed in a systematic and sequential manner until the full sample size has been obtained. At the initial trial visit, researchers will explain the study protocol to possible participants and obtain informed consent. Those screening positive for HTN and not willing to participate in the study will be referred for usual care.

Intervention Condition

After obtaining informed consent, a trained community health worker will introduce the DVD to the patient, explaining its purpose and how to use the DVD. Participants will view each intervention DVD installment at their local health center first and then engage in a post-media interview and problem-solving session with a community health worker. After the clinic viewing, the patient will take the DVD home for subsequent review and sharing with family and friends. At three months after randomization, the second installment of the DVD will be delivered for home viewing. After the second viewing, a study visit will be scheduled for a "post-media" interview and re-measurement of BP by a trained community health worker.

Comparison Condition

Participants randomized to the comparison condition will receive DVDs with only didactic material about common non-communicable diseases (e.g. diabetes, heart disease, chronic lung disease–"Learn More") but without HTN related stories. Otherwise, the treatment and study assessments for the intervention and comparison groups will be identical. The comparison group will receive usual medical care through the CHC.

Translation of Survey Instruments

We recognize the complexity involved in cross-cultural adaptations of behavioral interventions and in translating survey instruments and data collection protocols. In adapting the study protocols and translating the data collection instruments into Vietnamese, we will follow the set of best practices developed by the United States Census Bureau.[26] According to this protocol, translation will be accomplished by a translation team, with multiple versions prepared in parallel followed by team meetings to reconcile differences.

Our translation team will consist of the two Co- Principal Investigators (PI) from Vietnam who are fluent in the language, the PIs in the US, who bring important expertise about HTN and the intervention approach, and an expert in psychometrics. The translation process involves the following iterative steps: prepare, translate, pretest, revise, and document. In the preparation phase, the translation team will be given all relevant material to understand the purpose of the study, the intended audience, and the data collection modality and setting; translation by the Vietnam Co-Principal Investigators will proceed in parallel, with differences reconciled at team meetings. The pretesting phase will consist of cognitive interviews with five participants drawn from the local community. Cognitive testing will identify constructs specific to the Vietnamese language and culture so that appropriate adjustments may be made to ensure cross-cultural equivalence.[27] Several cycles of revision will be accomplished at full committee meetings conducted in person and by Internet video link. Each step in the process will be carefully documented. The ultimate aims of the translation process will be to produce a product that is reliable with semantic equivalence, technical accuracy and textual completeness, reads with fluency with a natural flow in Vietnamese, is appropriate to the literacy level of the intended audience, and is appropriate in style, tone, and degree of formality.

Data collection and management

Data will be collected at the time of baseline trial enrollment and subsequent follow-up visit at the participants' local CHC 3 months after randomization. Each study participants BP will be measured at each study visit according to a standardized protocol developed by the WHO. Trained community health workers will use a calibrated Omron automated monitor

to measure participant's BP, and the average of the last 2 of 3 readings will be entered into the database. Height and weight will be measured in the absence of shoes and heavy clothing while waist and hip size will be measured by placing the tape horizontally around the smallest part of the waist and the widest portion of the hips, respectively. Information on patients' use of traditional medicine for managing their HTN will also be collected..

Trained research nurses at each of the local CHCs will collect data on study participants socio-demographic factors and CVD risk factors including tobacco use, alcohol consumption, salt intake, and physical activity using the WHO STEPS questionnaires, which have been validated and used in previous studies examining risk factors for chronic diseases among rural Vietnamese adults.[28-31] We will ascertain self-reported engagement with the DVDs, including total viewing minutes, specific segments that were viewed, and whether the DVD was shared with family or friends. "Transportation" is a formally validated concept measuring absorption into the video narrative that has been linked to intervention effectiveness.[20, 21] Semi-structured interviews with the intervention group will solicit suggestions for refining the intervention before the larger trial. For example, participants will be asked to elaborate on what motivated or hindered their engagement with the intervention.

Data will be directly entered into a secure, password-protected, internet-enabled, laptop computer. The computer software will have a comprehensive set of built-in quality checks, such as mandatory completion of critical fields and out-of-range flags. After each instrument has been translated, the surveys will be uploaded into REDCap, an Internet-enabled database developed by the NIH Clinical and Translational Science Award program, which was successfully used in our previous work and which is available at no cost.

Study Outcomes

Feasibility outcomes include recruitment, retention, engagement, and treatment fidelity. Recruitment rates will be calculated from the number of patients approached and reasons for ineligibility and non-participation. We will record the number and reasons for failure to complete the follow-up assessment. Intervention engagement, which will be mainly ascertained from the patient survey as described above, includes time spent watching the

DVD, satisfaction with the viewing experience, and “transportation” into the story line. Treatment fidelity will be determined by the Vietnam Co-PIs directly monitoring 20% of all study enrollment and follow-up visits and completing a fidelity checklist which will be carefully documented. Exploratory outcomes will include levels of systolic and diastolic BP and the proportion of participants with controlled BP defined as in JNC-8 (≤140/90 mmHg for all individuals –a different cut-off will be used for those patients ≥60 years old and do NOT have diabetes and do NOT have chronic kidney disease for whom BP should be ≤ 150/90 mmHg) at three months after randomization.

The recruitment and retention rates of study participants will be calculated for each follow up visit in order to implement the intervention in a timely manner. Given our previous survey data about an individual’s willingness to participate in a clinical trial of HTN conducted at this study site, these rates are anticipated to be greater or equal to 80%. Examination of our final recruitment and retention rates will allow us to focus on specific aspects of the study protocol that need to be revised. Patient’s engagement and treatment fidelity will be collected through a questionnaire survey. Based on patient’s feedback, we will revise the intervention accordingly. We anticipate that changes in BP from baseline enrollment would be 2 mmHg or greater among those in the intervention than in the control group. Based on these findings, we will carry out sample size estimates to have adequate power to detect meaningful between group differences in our pre-specified major trial endpoints.

To prevent/minimize losses to follow up and missing data, one week before the scheduled follow-up visit local staff will contact participants by phone as a reminder or by a home visit. Since the communes are small, it is easy to visit participant’s homes. For participants who miss the follow up visits, study staff will come to their homes to interview and measure their BP within 2 weeks of a scheduled follow-up visit. During the course of the study, local staff will call participants every 2 weeks to find out if participants need any technical support for using DVD players, and encourage them to view the standardized DVDs more frequently to improve their adherence to the study intervention. Data collection forms are designed to be relatively short, straight forward, and culturally adapted which will

limit respondent burden and inconvenience. Finally, participants will receive a DVD player at no cost at the beginning of the study and an Omron BP monitor when they finish the study to support their initial and continued participation in the study.

Data Analysis Plan

Baseline characteristics of the intervention and usual care groups will be summarized using standard descriptive statistics. We will examine extent and mechanisms of missingness in data on each measure including participant satisfaction, recruitment, retention rates, baseline assessment, and BP. We will report numbers and reasons for recruitment and retention using a CONSORT diagram[32, 33] and will report results consistent with the CONSORT-EHEALTH checklist.[34] We will conduct a directed content analysis[35] of the open-ended questions to elaborate on the quantitative measures of engagement and to identify themes of satisfaction and suggestions for improvement.

Given the feasibility nature of this study, we will have limited ability to test hypotheses of intervention effectiveness. However, we will compare the distributions of systolic and diastolic BP and rates of BP control between the intervention and comparison groups. We will examine outcome variability within and between patients over time to inform the sample size calculations for the future trial. We will calculate the intraclass correlation coefficient (ICC) that accounts for the nesting of patients within commune.[36-39]

Sample Size

Leon, Davis, & Kraemer state that "power analyses should not be presented in an application for a pilot study that does not propose inferential results".[40] Instead, we based our sample size on accepted practice for pilot studies, the considerable experience of the research team, and practical considerations.[40, 41]

Timeline

The proposed study will last two years. During the initial 9 months we will develop the storytelling intervention; 12 months will be subsequently devoted to conducting the feasibility trial. The final three months will be devoted to data analysis, report and manuscript writing,

and planning the subsequent cluster-RCT.

Abbreviations

CVD: Cardiovascular disease

HTN: hypertension

NCD: Non-communicable disease

WHO: World Health Organization

BP: Blood pressure

RCT: Randomized controlled trial

CHC: Community health center

DVD: Digital video disc

CSI: Culturally Sensitive Intervention

PI: Principal Investigator

JNC: Joint National Committee

CONSORT: Consolidated Standards of Reporting Trials

References

1. Health Statistics Year Book 2006. Hanoi, Vietnam: Ministry of Health2007.

2. Health Statistics Yearbook 2000. Hanoi, Vietnam: Ministry of Health2001.

3. Jamison DT, Breman JG, Measham AR, Alleyne G, Claeson M, Evans DB et al. Disease Control Priorities in Developing Countries. Second Edition. Washington DC 20433: World Bank Publications; 2006.

4. Hoang VM, Dao LH, Wall S, Nguyen TK, Byass P. Cardiovascular disease mortality and its association with socioeconomic status: findings from a population-based cohort study in rural Vietnam, 1999-2003. Prev Chronic Dis. 2006;3(3):A89.

5. Vos T. A personal communication regarding to unpublished Burden of Disease study results of Developing Evidence Base for Health Policy in Vietnam (VINE) project 2009.

6. Son PT, Quang NN, Viet NL, Khai PG, Wall S, Weinehall L et al. Prevalence, awareness, treatment and control of hypertension in Vietnam-results from a national survey. Journal of human hypertension. 2012;26(4):268-80. doi:10.1038/jhh.2011.18.

7. Khai GP, Viet LN, Son TP, Quang NN, Yen BTN, Hung QN. Epidemiology survey of hypertension and its risk factors in Vietnam: Presentation at World Health Organisation's office-Hanoi, Vietnam. 2008.

8. Reducing Risks, Promoting Healthy Life. Geneva: World Health Organization2002.

9. Chobanian AV, Bakris GL, Black HR, Cushman WC, Green LA, Izzo JL et al. Seventh report of the joint national committee on prevention, detection, evaluation, and treatment of high blood pressure. Hypertension. 2003;42(6):1206-52.

10. Hoang V, M., Byass P, Wall S. Mortality from cardiovascular diseases in Bavi District, Vietnam. Scandinavian Journal of Public Health. 2003;31:26-31.

11. Ha DA, Goldberg RJ, Allison JJ, Chu TH, Nguyen HL. Prevalence, Awareness, Treatment, and Control of High Blood Pressure: A Population-Based Survey in Thai Nguyen, Vietnam. PloS one. 2013;8(6):e66792. doi:10.1371/journal.pone.0066792.

12. Tuan NT, Tuong PD, Popkin BM. Body mass index (BMI) dynamics in Vietnam. European Journal of Clinical Nutrition 2008;62(1):78-86.

13. Houston TK, Allison JJ, Sussman M, Horn W, Holt CL, Trobaugh J et al. Culturally appropriate storytelling to improve blood pressure. Annals of Internal Medicine. 2011;154(2):77.

14. Saha S, Beach MC, Cooper LA. Patient centeredness, cultural competence and healthcare quality. Journal of the National Medical Association. 2008;100(11):1275.

15. Rosenstock IM. The health belief model and preventive health behavior. Health Educ Behav; 1974.

16. Slater MD, Rouner D. Entertainment—education and elaboration likelihood: Understanding the processing of narrative persuasion. Communication Theory. 2002;12(2):173-91.

17. Ogedegbe G. Barriers to optimal hypertension control. Journal of clinical hypertension. 2008;10(8):644-6.

18. Borzecki AM, Oliveria SA, Berlowitz DR. Barriers to hypertension control. American heart journal. 2005;149(5):785-94. doi:10.1016/j.ahj.2005.01.047.

19. Hill MN, Sutton BS. Barriers to hypertension care and control. Current hypertension reports. 2000;2(5):445-50.

20. Green MC. Transportation into narrative worlds: The role of prior knowledge and perceived realism. Discourse Processes. 2004;38(2):247-66.

21. Green MC, Brock TC. The role of transportation in the persuasiveness of public narratives. J Pers Soc Psychol. 2000;79(5):701-21.

22. National survey on utilization of phone, Internet and television. Hanoi, Vietnam: Ministry of Information and Communications2011.

23. Nielsen J, Mack R. Usability Inspection Methods. New York: John Wiley & Sons; 1994.

24. Kushniruk AW, Patel VL, Cimino JJ. Usability testing in medical informatics: cognitive approaches to evaluation of information systems and user interfaces. Proc AMIA Annu Fall Symp. 1997:218-22.

25. Kushniruk AW, Patel VL. Cognitive computer-based video analysis: its application in assessing the usability of medical systems. Medinfo. 1995;8 Pt 2:1566-9.

26. Pan Y, de la Puente M. Census Bureau Guideline for the Translation of Data Collection Instruments and Supporting Materials: Documentation on How the Guideline Was Developed. 2005.

27. Census Bureau Standard: Pretesting Questionnaires and Related Materials for Surveys and Censuses. 2003.

28. Minh HV, Byass P, Chuc NT, Wall S. Gender differences in prevalence and socioeconomic determinants of hypertension: findings from the WHO STEPs survey in a rural community of Vietnam. Journal of human hypertension. 2006;20(2):109-15. doi:10.1038/sj.jhh.1001942.

29. Pham LH, Au TB, Blizzard L, Truong NB, Schmidt MD, Granger RH et al. Prevalence of risk factors for non-communicable diseases in the Mekong Delta, Vietnam: results from a STEPS survey. BMC public health. 2009;9:291. doi:10.1186/1471-2458-9-291.

30. Ng N, Van Minh H, Tesfaye F, Bonita R, Byass P, Stenlund H et al. Combining risk factors and demographic surveillance: potentials of WHO STEPS and INDEPTH methodologies for assessing epidemiological transition. Scand J Public Health. 2006;34(2):199-208. doi:10.1080/14034940500204506.

31. Hoang VM, Byass P, Dao LH, Nguyen TK, Wall S. Risk factors for chronic disease among rural Vietnamese adults and the association of these factors with sociodemographic variables: findings from the WHO STEPS survey in rural Vietnam, 2005. Prev Chronic Dis. 2007;4(2):A22.

32. Altman DG, Schulz KF, Moher D, Egger M, Davidoff F, Elbourne D et al. The revised CONSORT statement for reporting randomized trials: explanation and elaboration. Annals of internal medicine. 2001;134(8):663-94.

33. Moher D, Hopewell S, Schulz KF, Montori V, Gotzsche PC, Devereaux PJ et al. CONSORT 2010 explanation and elaboration: updated guidelines for reporting parallel group randomised trials. Bmj. 2010;340:c869. doi:10.1136/bmj.c869.

34. Eysenbach G. CONSORT-EHEALTH: improving and standardizing evaluation reports of Web-based and mobile health interventions. J Med Internet Res. 2011;13(4):e126. doi:10.2196/jmir.1923.

35. Hsieh HF, Shannon SE. Three approaches to qualitative content analysis. Qual Health Res. 2005;15(9):1277-88. doi:10.1177/1049732305276687.

36. Adams G, Gulliford MC, Ukoumunne OC, Eldridge S, Chinn S, Campbell MJ. Patterns of intra-cluster correlation from primary care research to inform study design and analysis. Journal of clinical epidemiology. 2004;57(8):785-94. doi:10.1016/j.jclinepi.2003.12.013.

37. Campbell MK, Fayers PM, Grimshaw JM. Determinants of the intracluster correlation coefficient in cluster randomized trials: the case of implementation research. Clinical trials. 2005;2(2):99-107.

38. Murray DM, Pals SL, Blitstein JL, Alfano CM, Lehman J. Design and analysis of group-randomized trials in cancer: a review of current practices. Journal of the National Cancer Institute. 2008;100(7):483-91. doi:10.1093/jnci/djn066.

39. Murray DM, Varnell SP, Blitstein JL. Design and analysis of group-randomized trials: a review of recent methodological developments. American journal of public health. 2004;94(3):423-32.

40. Leon AC, Davis LL, Kraemer HC. The role and interpretation of pilot studies in clinical research. J Psychiatr Res. 2011;45(5):626-9. doi:10.1016/j.jpsychires.2010.10.008.

41. Kraemer HC, Mintz J, Noda A, Tinklenberg J, Yesavage JA. Caution regarding the use of pilot studies to guide power calculations for study proposals. Arch Gen Psychiatry. 2006;63(5):484-9. doi:10.1001/archpsyc.63.5.484.
